# Mitigating Human–Wild Boar Conflicts: A Review of Integrated Strategies and Future Directions

**DOI:** 10.3390/ani16142142

**Published:** 2026-07-10

**Authors:** Zhiming Xu, Ke Rong, Minghai Zhang

**Affiliations:** College of Wildlife and Protected Area, Northeast Forestry University, Harbin 150040, China; 20032575@nefu.edu.cn

**Keywords:** human–animal conflict, wild boar, population control, physical protection, community engagement

## Abstract

Wild boar populations have expanded globally due to factors such as habitat restoration, climate change, and adjustments in hunting policies. This expansion has led to agricultural losses, threats to public safety, disease transmission, and ecological damage, thereby exacerbating human–animal conflicts. Current strategies for mitigating conflicts between wild boars and humans face challenges, including insufficient assessment of long-term control effects and limitations in population dynamics monitoring technologies. This paper reviews mitigation strategies for global wild boar conflicts from ecological, economic, and social perspectives and provides a comprehensive evaluation for their effectiveness. Existing mitigation strategies include population control (hunting management and reproductive regulation), habitat management (landscape barriers and food resource regulation), physical protection (electric fences and multimodal deterrence devices), and community engagement mechanisms (ecological compensation and participatory management). The effectiveness of these strategies varies significantly across regions. Global case studies on human–wild boar conflicts demonstrate that multidimensional collaborative governance has achieved notable success in mitigating these conflicts. For example, dynamic management systems based on intelligent monitoring and community participation can effectively reduce the incidence of wild boar conflicts. Given the limitations of the current single-indicator evaluations, this paper proposes a comprehensive evaluation framework encompassing “strategy effectiveness, cost, and sustainability”. The application of synergistic multi-strategy approaches produces significant synergistic effects, exhibiting nonlinear superposition characteristics. Therefore, future efforts should integrate intelligent early warning systems and policy reforms to develop an adaptive social–ecological coupling framework.

## Simple Summary

Wild boar populations are increasing sharply worldwide, leading to more frequent conflicts with humans. These conflicts result in damage to agricultural crops, threaten public safety, transmit diseases to livestock and humans, and disrupt natural ecosystems. This paper reviews various strategies employed to manage wild boar populations and mitigate their impacts, including hunting, fencing, habitat management, and community-based compensation programs. Our analysis indicates that no single approach is universally effective; rather, the most successful strategies combine multiple methods tailored to local conditions. Integrating techniques such as regulated hunting, electric fencing, and community engagement yields better outcomes than relying on any single method alone. We also highlight significant gaps in current knowledge, particularly the scarcity of long-term studies evaluating the effectiveness of these measures over extended periods. These findings offer practical guidance for policymakers, farmers, and wildlife managers seeking sustainable solutions to balance wild boar conservation with human interests.

## 1. Introduction

The wild boar (*Sus scrofa*), also known as the wild swine, common wild pig, Eurasian wild pig, or simply wild pig, is a suid species native to extensive regions of Eurasia and North Africa. It has also been introduced to the Americas and Oceania. In their native Eurasian range, wild boar groups are also commonly called ‘herds’ or ‘sounders’ (the latter term is also used in the United States). With exponential population growth and a significant expansion of its global distribution in recent years [1], the wild boar has become one of the most widely distributed mammals worldwide and the most widespread suiform. In Zhejiang Province, China, the density of wild boars has reached approximately 1.8 individuals per km^2^, with a total population of exceeding 110,000 [2]. Similarly, in Europe, wild boar densities have also reached historical peaks in regions of Spain and France [3]. Their distribution now extends from natural habitats to urban fringes and agricultural areas, including the urban zones of Barcelona and Seoul [4]. The expansion of wild boar populations not only threatens biodiversity but also exacerbates human–animal conflicts.

The drivers of wild boar population growth are complex and multifaceted [5], primarily encompassing the following three aspects: (1) Habitat restoration and ecological engineering: The implementation of global ecological restoration projects, such as converting farmland back to forest and expanding protected areas, has significantly improved the quality of wild boar habitats. Meanwhile, the “blurring of ecological-agricultural boundaries” during urbanization has forced wild boars to invade areas of human activity, intensifying human–animal conflicts [6]. For example, China’s Three-North Shelter Forest Program and Europe’s Forest Regeneration Program have provided abundant food resources and shelter for wild boars [7]. Additionally, vegetation restoration in Zhejiang Province has expanded the wild boar’s range to 90% of counties [8]. (2) Impact of climate change: Climate warming extends the breeding season of wild boars and reduces the winter mortality rate of their young. Moreover, annual precipitation and temperature seasonality are significantly positively correlated with wild boar density. For instance, increased spring precipitation in Catalonia, Spain, directly promotes wild boar population growth [3]. (3) Adjustments to hunting policies: Since the late 20th century, the strengthening of biodiversity conservation policies worldwide—including multiple revisions to the Wildlife Protection Law of the People’s Republic of China—has restricted human hunting activities of wild animals [9]. However, issues such as inaccurate data monitoring, imperfect quota allocation mechanisms, and insufficient enforcement supervision in hunting quota management systems in some countries have limited effective control of wild boar populations, further exacerbating the loss of control over their distribution.

It is well established that wild boars are highly ecologically adaptable worldwide, characterized by extremely high reproduction rates—up to two litters per year in their native ranges, with 4 to 8 piglets per litter—although year-round breeding can occur in introduced regions such as the United States [10]. They also exhibit omnivorous behavior [11] and demonstrate a high tolerance for human activity. These traits make them a species frequently involved in complex human–wildlife interactions in both urban and agricultural landscapes [3]. While wild boars are integral components of their native ecosystems and can positively influence biodiversity, their overabundance and range expansion can significantly alter plant community structures through soil disturbance. Furthermore, their increasing presence in human-dominated areas leads to negative interactions that often trigger human–human conflicts regarding their management. Consequently, fostering human–wild boar coexistence and managing these interactions have become prominent challenges in global wildlife management. The primary consequences of their overabundance include agricultural damage, public safety concerns, veterinary and public health risks, and ecological alterations [7,12,13,14]. These consequences are manifested as:

(1) **Agricultural losses.** Ground-rooting behavior (foraging for subterranean plant parts), trampling, and group movements (with typical herd sizes of 20–30 individuals) are the primary mechanisms driving agricultural damage [12]. These behaviors mainly affect staple and cash crops such as corn, rice, sweet potatoes, and peanuts [7,14]. Consequently, crop depredation and the resulting economic losses constitute the primary drivers of negative human–wild boar interactions [15]. Such agricultural damage has become a significant factor threatening local food security and reducing farmers’ incomes [16]. In China, wild boars have expanded their range to 28 of the country’s 34 provincial-level administrative regions, with farmland in Zhejiang, Jilin, and Shaanxi provinces experiencing the most severe damage [13,14,17,18]. Historical data from 1956 to 2021 reveal a sharp upward trend in crop depredation in China; the average annual crop loss area surged from 40 hectares (with economic losses of approximately 232,500 CNY/year) during 1956–2000 to 1520 hectares (amounting to 25.06 million CNY/year) between 2001 and 2021 [19]. Similarly, in Nepal, wild boars around Shuklaphanta National Park and Dhorpatan Hunting Reserve caused substantial damage to rice and potatoes, resulting in an estimated total crop loss of 87,035 kg and corresponding economic losses of $26,389 USD [20]. In Italy, the wild boar population doubled from 500,000 in 2010 to over one million in 2021 and continues to grow. This demographic explosion has led to persistent agricultural damage, predominantly affecting vineyards, corn, and hazelnuts [21]. For instance, between 2006 and 2012, 221 incidents of crop damage were recorded in northeastern Sardinia alone, totaling €484,000 in economic losses [21].
(2) **Public safety threats.** The expansion of wild boars into human-dominated landscapes has led to an increased frequency of human–wild boar encounters, posing significant threats to public safety. Currently, over 60 cities worldwide report wild boar overabundance, which frequently results in severe wildlife–vehicle collisions (WVCs) [22]. In Germany, WVCs pose a substantial threat to public safety, causing approximately 2500 human injuries and up to 20 fatalities annually, with wild boars being among the most frequently involved species [22]. Similarly, in Spain, 74,600 wildlife–vehicle collisions were recorded between 2006 and 2012, with 79% of these incidents involving wild boar. Furthermore, reported incidents of negative human–wild boar interactions in Nanjing, China, increased exponentially from 2000 to 2022 [8]. Beyond vehicle collisions, wild boars can also threaten public safety through occasional aggressive behavior, including direct attacks on humans (particularly during mating season or when defending offspring), as well as damage to infrastructure and property.
(3) **Public health risks**. Wild boar serves as natural reservoirs for numerous dangerous pathogens, including severe epizootic viruses such as pseudorabies virus and African Swine Fever Virus (ASFV), as well as zoonotic bacteria including *Brucella suis*. This reservoir status significantly elevates the risk of cross-species transmission [14]. During ASFV outbreaks, wild boar populations act as key epidemiological vectors for trans-regional transmission, as their movements facilitate the spread of the virus across geographical barriers. Furthermore, interactions between wild boar and domestic pigs frequently trigger periodic outbreaks of highly contagious diseases such as classical swine fever virus (CSFV), establishing a bidirectional wildlife-livestock transmission cycle [23]. Consequently, the ongoing demographic and spatial expansion of wild boar not only poses a severe threat to agricultural biosecurity and the livestock industry but also substantially increases the risk of zoonotic spillover into human populations.
(4) **Ecological impact.** While wild boars play fundamental ecological roles in their native ranges—such as facilitating soil aeration and seed dispersal—their overabundance or introduction as an exotic species can lead to severe ecological degradation. As opportunistic omnivores, wild boars forage extensively on both above-ground vegetation and subterranean plant parts. Their characteristic ground-rooting behavior severely disrupts soil strata and damages root systems, accelerating soil erosion, hindering seedling regeneration, and compromising the structural integrity of forest and grassland habitats [23,24]. Furthermore, wild boars act as active predators of small vertebrates and invertebrates and compete directly with sympatric native species [16]. These trophic interactions collectively disrupt the structure and dynamics of local biological communities. Extensive rooting also alters soil microbial communities, leading to profound impacts on sensitive ecosystems [24]. For instance, where introduced as an exotic species in California, USA, wild boar activity has been linked to a 40% reduction in ectomycorrhizal fungal diversity, directly threatening oak seedling survival. Even within their native European range, localized population explosions can disrupt trophic balances; studies indicate that for every increase in wild boar density by one individual per km^2^, the foraging efficiency of the European bison decreases by 22% [24].

In this study, we present a review of human–wild boar conflict and its mitigation strategies based on published literature. Literature searches were conducted using keyword combinations such as “human–animal conflict,” “wild boar,” “mitigation strategies,” and “population control,” covering databases including Web of Science and China National Knowledge Infrastructure (CNKI). The final inclusion criteria for the literature were: (1) empirical studies addressing wild boar conflict mitigation strategies; (2) provision of quantitative effect evaluation data; (3) publication between 2000 and 2025 (to focus on recent advances; earlier foundational studies before 2000, while valuable, are not the primary focus of this review); (4) exclusion of purely theoretical models and studies without field validation. Literature growth can be divided into four stages by correlating policy events with technological breakthroughs, as shown in Table 1. Studies published between 2000 and 2010 focused on ecological surveys (such as wild boar population density monitoring) and the assessment of basic protective measures (such as fencing). During the stable growth phase, research topics expanded to include reproductive regulation (such as GnRH vaccine trials) and habitat management (such as isolation zone design). Subsequently, literature growth synchronized with policy interventions and technological advancements (e.g., AI warning systems and reproductive regulation), reflecting the research’s responsiveness to practical governance needs.

Existing research has primarily focused on individual wild boar management methods, such as hunting or fencing. However, the complexity of wild boar behavior and regional variations necessitate the development of comprehensive strategies [25]. Therefore, it is crucial to investigate the current status of strategies aimed at mitigating wild boar conflicts. From ecological, social, and economic perspectives, this study reviews existing mitigation approaches and proposes an integrated management framework based on “social–ecological coupling” for addressing conflicts between wild boars and humans. This framework incorporates a closed-loop system of “monitoring–early warning–intervention–assessment”, emphasizes collaboration among sectors such as forestry, agriculture, and public health, encourages public participation, and seeks to balance species conservation with human interests. Here, “social–ecological coupling” refers to the interdependent feedback mechanisms among wild boar population dynamics, habitat conditions, and human management decisions. This review explores potential pathways toward sustainable human–wild boar coexistence.

## 2. Existing Mitigation Strategies

### 2.1. Preventive Strategies

#### 2.1.1. Physical Deterrence Systems

Electric fencing systems induce conditional avoidance responses in wild boars through painful stimuli generated by pulsed currents, making them one of the most reliable physical protection methods currently available. Long-term controlled experiments conducted in Hunchun, China, demonstrate that electric fences provide significantly longer effective protection periods compared to other visual or auditory deterrents. Research indicates that 2-wire and 3-wire electric fence systems can offer continuous protection throughout the critical 30-day maize maturation period, substantially delaying wild boar intrusion and effectively reducing crop loss rates [26]. However, the effectiveness of electric fences is limited by maintenance costs and topographical challenges. Despite their high initial investment and installation difficulties in complex terrains, these systems deliver a superior long-term cost–benefit ratio compared to traditional fencing. Nonetheless, they require a continuous power supply and ongoing maintenance, which complicates their implementation in remote or underdeveloped areas [26].

Dynamic deterrence devices employ sensory stimuli such as sound, light, and olfaction to disrupt wild boar behavior, offering advantages including low cost and flexible deployment. However, their primary limitation is the habituation of the target species [26]. Visual deterrents, particularly red solar blinkers, have demonstrated significant short-term repellent effects (approximately 14–32 days) and effectively reduce farmland intrusion events. Nevertheless, prolonged exposure leads wild boars to adapt to the light stimulus, resulting in a rapid decline in protective efficacy [26]. Similarly, auditory deterrents, such as playbacks of predator sounds (e.g., tiger roars) or conspecific calls, can elicit a startled response. However, a pilot study in Germany found that without an associated real predation risk, simple acoustic stimuli are insufficient to sustain long-term avoidance behavior [27].

Olfactory and integrated deterrents have been investigated for the short-term mitigation of wild boar activity. A German study demonstrated that measures such as blue LED flashers, aluminum tapes, and specific chemical agents (e.g., Hukinol™) reduced the likelihood of wild boars contacting attractants to one-third of that observed in the control group. This indicates their potential utility in emergency situations, such as temporary carcass isolation in African Swine Fever-endemic areas. However, their long-term effectiveness in large-scale farmland remains uncertain [27]. To address the primary challenge of habituation, case studies in Zhejiang, China, suggest that relying on a single deterrent modality is often ineffective. Instead, combining auditory and visual stimuli or dynamically adjusting stimulation patterns based on wild boar activity rhythms (e.g., crepuscular peaks) can delay the habituation process [2].

#### 2.1.2. Habitat Management

Landscape patterns are key factors influencing the spatial distribution of human–wild boar conflicts. Multi-scale risk modeling studies indicate that the risk of wild boar damage to farmland is closely associated with landscape heterogeneity. In Jilin Province, China, the forest–farmland ecotone—particularly areas with gentle slopes—represents a high-risk zone for conflicts, with damage hotspots primarily concentrated within approximately 150 to 250 m from the forest edge [28,29]. Based on these findings, risk can be mitigated by optimizing landscape structure, such as establishing buffer zones along forest edges. However, physical barriers (e.g., fences) without accompanying management strategies may provoke behavioral adaptations in wild boars, such as seeking alternative intrusion routes [30].

Wild boar foraging behavior is strongly influenced by their perception of risk. Comparative research conducted in Israel revealed that wild boars in agricultural areas, where hunting pressure is high, exhibit pronounced risk-avoidance behaviors and heightened vigilance toward novel objects (e.g., feeding devices). In contrast, wild boars in urban areas, which experience minimal human interference, display low risk perception and habituate more readily [30]. This suggests that reinforcing fear by increasing signs of human activity or implementing legal hunting pressure can alter wild boar foraging patterns and reduce their reliance on specific farmlands [28,30]. Although supplementary feeding is often proposed as a method to divert wild boar feeding pressure away from crops, long-term monitoring data from Europe highlight potential risks. Excessive food supplementation may lead to a reduction in wild boar home range sizes and artificially increase population density, thereby exacerbating local conflicts [31]. Therefore, any strategy involving food resource regulation must be based on rigorous population dynamics monitoring. Furthermore, based on risk prediction models, planting crops that are unpalatable to wild boars or have low economic value and high resistance to damage (e.g., Chinese herbal medicines, thorny shrubs) in high-risk areas along forest edges can serve as an effective spatial avoidance strategy [29].

#### 2.1.3. Intelligent Monitoring Systems

Intelligent monitoring technology is increasingly serving as the scientific foundation for conflict management, with its core focus on accurately understanding population dynamics and activity patterns. Infrared camera networks, exemplified by a large-scale monitoring system comprising 1271 units established in Zhejiang Province, China, have facilitated density estimation (approximately 1.77 individuals/km^2^) and identification of conflict hotspots (e.g., Lishui and Hangzhou) using the Random Encounter Model. This approach has significantly reduced estimation errors and supported macro-level management decisions [2]. Concurrently, the use of bio-loggers, such as tri-axial accelerometers, has enhanced behavioral analysis. For instance, research conducted in the Czech Republic reconstructed wild boar movement paths and revealed a high tolerance to specific anthropogenic disturbances (e.g., vehicle and chainsaw sounds), with most individuals remaining at rest despite these disturbances. This challenges the conventional view that human activity inevitably displaces wild boars and suggests that limited human interventions (e.g., carcass removal) during African Swine Fever control may not provoke widespread dispersal [32].

Furthermore, long-term monitoring has revealed distinct spatiotemporal activity patterns in areas lacking large predators or hunting pressure (e.g., Zhejiang). In these regions, wild boars display pronounced diurnal tendencies, with activity peaks at 7:00, 11:00, and 16:00 in zones experiencing high damage. This deviates from the traditionally assumed nocturnal behavior, confirming their diurnal activity patterns in these areas. Understanding these rhythms is crucial for scheduling preventive measures ahead of peak activity periods [2].

### 2.2. Control Strategies

The core conflict in human–wild boar interactions stem from the competition for resources driven by population growth pressure and limited habitat availability. Ecological studies indicate that wild boar populations possess significant compensatory reproductive capacity, making it challenging to maintain ecological carrying capacity through natural regulatory mechanisms alone [31]. In this context, implementing scientifically based population control has become a crucial strategy for mitigating these conflicts. Based on factors such as duration of effect, technical mechanisms, and intervention reversibility, control strategies can be categorized into two main types: hunting regulation and reproductive control. Although wild boars are managed using various control methods worldwide—including trapping, aerial shooting, and toxicants in some introduced ranges—this review primarily focuses on methods applied within native Eurasian range. Examples from introduced ranges (e.g., California, USA) are included only for comparative illustration and are explicitly identified as such.

#### 2.2.1. Hunting Control

Hunting is currently the primary method for controlling wild boar populations worldwide; however, its effectiveness is limited by several factors, including the number of hunters, hunting techniques, and the behavioral adaptability of wild boars. Long-term monitoring data from Europe indicate that, although hunting remains the main cause of wild boar mortality, European wild boar populations have continued to increase over the past three decades, with annual growth rates significantly exceeding 1. This trend is primarily attributed to a stagnation or decline (18% decrease) in the number of hunters, resulting in reduced hunting pressure. The current recreational hunting model is insufficient to curb population growth and may even trigger compensatory reproductive mechanisms, such as earlier sexual maturity in juvenile females, due to changes in population structure, ultimately leading to population rebounds [31].

The spatial distribution of hunting activities significantly influences wild boar behavior. Research conducted in Catalonia, Spain, confirmed that when surrounding areas are open to hunting, wild boars congregate in large numbers within nature reserves where hunting is prohibited, seeking refuge. Although this aggregation increases population density and management pressure within the reserve, it also creates opportunities for targeted control. Studies indicate that implementing occasional control drives within reserves can effectively remove aggregated individuals, with effects lasting at least 45 days [33].

Research conducted in Lower Saxony, Germany, examined the efficiency of various hunting methods. Although individual hunting—particularly at bait sites—remains the most commonly used approach, its effectiveness is limited by the willingness and skill of individual hunters. In contrast, “driven hunts” conducted across adjacent hunting grounds have proven to be a more efficient means of population control, significantly increasing the harvest per man-hour. Therefore, enhancing cooperation and coordination among hunters across regions and promoting driven hunts are essential strategies for improving hunting control efficiency [34].

Hunting pressure can also influence wild boar spatial utilization patterns by altering their perception of risk. A study conducted in Israel found that wild boars in agricultural areas subjected to long-term, high-intensity hunting pressure exhibit pronounced risk-avoidance behaviors and heightened vigilance toward novel objects. In contrast, wild boars in urban or protected areas, where human interference is minimal, demonstrate lower risk perception. This suggests that maintaining appropriate hunting pressure serves not only as a direct lethal control method but also as an effective fear-based tool that discourages wild boars from occupying specific high-risk areas [30].

#### 2.2.2. Reproductive Control

Given the limitations of hunting in certain areas (e.g., urban edges or protected zones), non-lethal reproductive control technologies are considered promising alternatives. Reproductive control primarily disrupts physiological reproductive processes through biological interventions, such as immunocontraceptive vaccines. Although theoretically feasible, practical application currently faces significant challenges. A review conducted in Europe highlights that existing fertility control technologies, such as GnRH vaccines, require injection following individual capture, resulting in extremely high operational costs and making large-scale implementation in wild populations difficult [31].

Furthermore, the effectiveness of reproductive control is limited by its coverage. If interventions cannot reach the majority of the population, their impact on curbing population growth will be minimal. Additionally, the literature [32] indicates that wild boars exhibit a high tolerance to anthropogenic disturbances, making capture and intervention operations more challenging than anticipated. This method is primarily restricted to small-scale, high-value, specific-area trials and does not yet have the capacity to replace hunting as the primary control method [31].

### 2.3. Compensation Strategies

#### 2.3.1. Ecological Compensation Systems

The economic loss compensation mechanism serves as a fundamental component of social governance aimed at mitigating negative interactions between humans and wild boars. The scientific rigor of its system design directly influences farmers’ willingness to support conservation efforts. Wild boar crop damage poses a significant threat to rural livelihoods. Research conducted in the Three Gorges Reservoir area of China indicates that wild boar damage reduced maize and sweet potato yields by approximately 25% and 28%, respectively, resulting in substantial annual economic losses. This damage was primarily concentrated within 150 m of the forest edge [29]. Similarly, around Shivapuri Nagarjun National Park in Nepal, economic losses caused by wild boars accounted for roughly 9% of farmers’ expected profits, representing a major source of conflict [35]. These findings underscore the importance of establishing effective compensation mechanisms to sustain farmers’ livelihoods.

Although compensation policies are necessary, their implementation often fails due to bureaucratic inefficiencies and a lack of transparency. Cases from central Italy demonstrate that, despite the adoption of both preventive measures and compensation schemes, the cumbersome procedures and prolonged waiting times (sometimes one to two years), combined with a lack of transparency, have fostered significant distrust between farmers and management departments, thereby exacerbating social conflicts. This indicates that simple economic payouts, without process optimization, are unlikely to effectively resolve underlying issues [36]. Research conducted in Fujian Province, China, revealed that the economic vulnerability of different types of farmers (such as specialized farmers and pure farmers) affected by wild boars varies significantly, highlighting the need for differentiated compensation policies [37]. Additionally, compensation mechanisms should incorporate incentive compatibility; for example, when determining compensation, whether farmers have implemented active protective measures should be considered to prevent dependency or moral hazard [35].

#### 2.3.2. Participatory Management

Cognitive differences among stakeholders and a lack of trust are significant social factors that intensify human–boar conflicts [38]. Establishing multi-party collaborative decision-making mechanisms can help resolve these deep-seated social contradictions. The essence of such conflicts often extends beyond the direct damage caused by wildlife to reflect underlying human social relations. Research indicates that social factors—such as risk perception, power inequality, and cultural beliefs—are often more fundamental drivers of conflict than the actual ecological damage. Ignoring these factors and relying solely on technical measures to reduce damage frequently results in the long-term failure of mitigation strategies [38].

The “Wild Boar Decision Simulation System” (WILD BOAR(D) GAME), developed by the Camargue Biosphere Reserve in France, provides a successful model of participatory management. This tool simulates wild boar behavior and management decision-making interactions through role-playing games involving hunters, farmers, and managers. Results demonstrated that the game not only facilitated knowledge sharing but also effectively reduced tensions, increasing the implementation rate of management measures from 62% to 89%. This evidence shows that such intermediary tools can effectively promote collective learning and consensus building at the local or community scale [39]. Their applicability to regional- or national-level governance remains to be tested. In Zhejiang Province, China, researchers combined damage surveys and infrared camera data to develop a conflict mitigation framework based on “damage–distribution–activity,” offering a scientific foundation for precise management at the county level—an example of scientific participatory management [2]. However, the implementation of participatory management is limited by the local social and institutional environment. For example, research in southern India revealed that conflict risk is influenced by multiple ecological factors, such as crop abundance and topography; effective community participation requires a thorough understanding of these local drivers and the adoption of targeted, community-based joint defense measures [40].

## 3. Case Analysis

Agricultural losses caused by wild boars worldwide amount to billions of dollars annually, with conflict events prevalent in developing countries [41]. The global issue of wild boar damage exhibits significant regional heterogeneity, and the effectiveness of management strategies depend heavily on the coupling mechanisms within the ecological–social system. Consequently, despite the variety of management measures implemented by different countries, the success of these strategies varies considerably due to regional characteristics, sociocultural factors, and differences in policy enforcement. Through cross-regional comparative analysis, this study reviews successful and unsuccessful cases from representative regions worldwide, uncovers the underlying logic and applicable boundaries of various governance models, and integrates a multidisciplinary perspective to establish a theoretical foundation for proposing a universal, integrated management framework for conflict mitigation. This framework aims to provide a scientific basis and practical guidance for developing sustainable conflict mitigation strategies.

### 3.1. Success Case

Successful global wild boar conflict governance practices are generally characterized by a three-dimensional synergy of technology, policy, and society. The core approach involves identifying key conflict nodes through scientific monitoring, reconstructing interest coordination mechanisms via institutional innovation, and ultimately enhancing the resilience of the governance networks through community empowerment. The following three representative cases illustrate the breakthrough application of ecological wisdom, game theory tools, and urban adaptive management in resolving human–animal conflicts.

(1) Zhejiang Province, China: *Synergistic Management Based on Ecological Monitoring and Community Engagement.* Zhejiang Province, which is a high-incidence area for wild boar conflicts in China, has developed a dynamic management model by integrating infrared camera monitoring technology with community participation mechanisms. The research team deployed 1271 infrared cameras between 2019 and 2023. Using the random encounter model, they estimated wild boar density at 1.8 ± 0.4 individuals/km^2^. Lishui, Hangzhou, and Jinhua were identified as core conflict areas [2]. The specific management strategies include: ① Spatial zoning management: designating mountainous areas with high vegetation coverage and low GDP as “active control zones,” prioritizing physical isolation (such as power grid fences) and habitat transformation (reducing crop planting density). ② Community compensation mechanism: the government established special funds to compensate farmers proportionally for crop losses while promoting high-resistance crops (such as konjac) to reduce economic dependence [42]. ③ Public education program: through village-level lectures and school courses to disseminate knowledge of wild boar behavior and reduce irrational panic [9]. As a result, the area affected by wild boar damage in Zhejiang Province decreased by 42%, and the public support rate reached 78% [2].
(2) France: *A Conflict-Mitigation Experiment Based on Game Theory.* The Camargue Biosphere Reserve in southern France employs role-playing games to facilitate stakeholder dialog. The research team designed the “Wild Boar Game” to simulate the dynamic balance among wild boar habitat selection, hunting policies, and agricultural losses [39]. Participants—including farmers, hunters, and policymakers—negotiated to develop management rules. The results demonstrated: ① A collaborative decision-making mechanism: The game fostered consensus on “hunting quotas” and “ecological compensation,” with 80% of participants supporting limits on hunting intensity to maintain population stability. ② A behavioral pattern shift: The failure rate of the deterrent devices in strong winds reached as high as 45% [43]. This case underscores the importance of evaluating technological applications in conjunction with local cultural adaptability.
(3) Seoul, South Korea: *Adaptive Management of Urban Ecosystems.* Seoul proposed a “gradient control strategy” by analyzing the relationship between wild boar activity patterns and urban land use. The study found that wild boars in the suburbs of Seoul are most active during sunrise and sunset, and they prefer abandoned farmland and low-density residential areas [44]. Management measures include: ① Blocking habitat connectivity by establishing 3 m wide shrubbery belts along the foothills to reduce the spread of wild boars into urban areas. ② Implementing a smart early warning system using AI cameras and vibration sensors to monitor intrusions in real time and send alerts via mobile phone apps [45]. ③ Regulating the food chain by introducing natural predators of wild boars, such as wolves, to restore ecological balance. This approach is still in the experimental verification stage but has shown significant results: wild boar sightings in Seoul’s urban area have decreased by 65%, and no serious personal injury reports have been recorded [44].

### 3.2. Unsuccessful Cases

In contrast to successful cases, governance failures often arise from the limitations of a single intervention logic. Examining failure experiences can offer valuable cautionary examples for understanding the vulnerability of the governance systems. The following cases reveal three critical flaws: structural deficiencies in institutional design that render compensation mechanisms ineffective; cultural disembedding of governance, which leads to tool failure; and the inherent contradictions in policy goals that further intensify conflicts between humans and wildlife.

(1) Shukrapanta National Park, Nepal: *Ongoing conflict persist due to the absence of effective compensation mechanisms.* In the communities surrounding Shukrapanta National Park in western Nepal, farmers’ economic losses have remained unresolved for an extended period. Studies indicate that wild boars damage approximately 8% of the annual crop yield, yet government compensation covers less than 30% of these losses [20]. The reasons for this failure include: ① Delayed policy implementation: The compensation application process is cumbersome, with an average processing time of up to six months, leading may farmers to abandon their claims. ② Unequal distribution of funds: Compensation funds are prioritized for tourist hotspots, while remote villages are neglected. This case demonstrates that a strategy relying solely on economic compensation, without community empowerment, is difficult to sustain.
(2) East Macedonia, Greece: *Insufficient public support due to the failure of non-fatal measures.* In Greece, efforts to promote sonic repellents and odor barriers in the eastern Macedonia region have failed due to low public participation. A survey revealed that only 12 percent of farmers regularly maintained the equipment, while 38 percent of hunters opposed restrictions on hunting [43]. The underlying issues stem from: ① Cultural cognitive differences—traditional hunting culture regards wild boars as “honorable prey” and resists non-lethal management. ② Inadequate technical adaptability—the failure rate of the repeater in strong winds reaches as high as 45% [43]. This case underscores the importance of evaluating technological applications in conjunction with local cultural adaptability.
(3) British Forest Dean, United Kingdom: *Policy contradictions intensifying human–animal confrontation.* The “rewilding movement” policy has encouraged the return of wild boars, but no population control plan was developed concurrently, leading to an escalation of conflicts. From 2015 to 2020, the annual growth rate of wild boar attacks was 22%, while public support plummeted from 68% to 31% [46]. The main reasons for the policy failure include: ① Ambiguous management authority, with jurisdictional conflicts between environmental protection departments and local governments over hunting licenses. ② Lack of scientific monitoring, as population estimation errors exceeds 40%, resulting in delayed control measures [46]. This case serves as a warning that ecological restoration projects must be accompanied by dynamic monitoring systems.

### 3.3. Case Comparison and Implications

The specific management objectives for each case are outlined at the beginning of their description in Section 3.1 and Section 3.2. The following common features pertain to the successful attainment of these stated goals. As shown in Table 2, through cross-case comparisons, this study uncovers the underlying mechanisms driving differences in governance effectiveness for wild boars and offers key insights for developing a universal governance framework. Successful cases exhibit a “three-dimensional resilient structure,” whereas unsuccessful cases reveal “governance system breakpoints.”

Three common features characterize successful governance: ① Adaptive governance scale, involving multi-scale nesting from ecosystems (Seoul) and administrative divisions (Zhejiang) to social networks (France); ② Integration of intervention logic to achieve a three-dimensional approach encompassing population regulation, damage mitigation, and cognitive management; ③ Construction of feedback mechanisms by establishing adaptive loops through intelligent monitoring (Seoul), participatory evaluation (Zhejiang), and game simulation (France). The transformation paths from these three successful cases to a universal framework can be summarized as follows: ① Geographical adaptation principle: Developing countries should prioritize building a “policy–community” dual-pillar model (as exemplified by Zhejiang), whereas developed countries can focus on collaborative innovation between technology and institutions (as seen in Seoul). ② Phased implementation strategy: Implement a “rapid response package” (including temporary compensation and emergency prevention and control) during the early stages of conflict, then transition to a “resilience building package” (including monitoring networks and community empowerment) in the medium to long term.

The failed cases all exhibit missing governance elements: policy coherence (Nepal), technical adaptability (Greece), and scientific rigor in monitoring (UK). In the Nepal case, the absence of a comprehensive institutional design covering the entire compensation process—application, assessment, and disbursement—rendered the policy merely symbolic. In Greece, the sonic drive failed under strong wind conditions, revealing that the technical solution must undergo a dual “technology–culture” fit assessment, integrating user cognition and environmental constraints into the design. In the UK case, a 40% error in population estimation caused delays in control measures, underscoring the necessity for monitoring systems to satisfy dual criteria of spatial-temporal continuity and data accuracy.

## 4. Evaluation of Strategy Effectiveness

To evaluate mitigation strategies for human–wild boar conflicts, we adopt the social–ecological systems (SES) framework. This framework recognizes that conflicts arise from both biophysical processes (e.g., population dynamics, habitat quality) and social factors (e.g., land-use policies, cultural perceptions). Accordingly, a strategy’s success depends on its ecological effectiveness and its social acceptance. Outcomes vary considerably by regional conditions and implementation contexts, leading to wide differences among current strategies in technical efficacy, cost-effectiveness, ecological impact, social acceptance, and sustainability. To compare these diverse strategies beyond single indicators, we apply the SES framework in the multidimensional assessment described in Section 4.1.

### 4.1. Construction of Strategy Effectiveness Evaluation Framework

We propose an “efficacy–cost–sustainability” (ECS) framework, which integrates the social–ecological systems (SES) perspective into five core dimensions for practical evaluation. Technical efficacy refers to a strategy’s direct impact on achieving primary goals, such as damage reduction rates and protection duration. Economic feasibility assesses the balance between implementation costs and benefits, emphasizing trade-offs—for example, the high initial cost of electric fences versus the low cost of bait crops. Ecological adaptability pertains to a strategy’s effects on species behavior and ecosystems, such as rapid habituation to deterrents or population growth resulting from excessive supplementary feeding. Social acceptability denotes the level of stakeholder support, which is crucial for implementation, as illustrated by differing public opinions on lethal control [36]. Management sustainability involves long-term operational stability and institutional reliance, including ongoing technical support for monitoring systems and the institutional trust necessary for participatory management [39]. We employ a mixed-methods approach, combining quantitative empirical data from the literature with qualitative assessments of less quantifiable aspects, such as social acceptance.

### 4.2. Multidimensional Evaluation and Analysis of Major Mitigation Strategies

#### 4.2.1. Preventive Strategies: Trade-Offs Between Efficacy, Cost, and Habituation

Preventive strategies involve trade-offs among efficacy, cost, and habituation. Electric fences demonstrate high technical efficacy; studies in Hunchun, China, show they provide effective protection for over a month and offer a better long-term cost–benefit ratio than traditional fences [26]. However, their high initial and maintenance costs limit economic feasibility, and wild boars may adapt by digging under fences or shifting their activity ranges [30]. In contrast, dynamic deterrents such as solar blinkers are low-cost and initially effective [26], but rapid habituation reduces their long-term effectiveness. Randomizing stimuli can delay habituation but increase operational complexity. Habitat and food management strategies, such as “bait crops,” are also low-cost and can divert wild boars to buffer zones [37]. Nevertheless, their ecological impact requires caution; excessive feeding can increase population density—the “feeding paradox”—rather than reduce conflicts [31]. Therefore, food resource regulation must adhere to strict thresholds. Shortened: Preventive strategies balance efficacy, cost, and habituation. Electric fences are effective long-term but costly and susceptible to behavioral adaptation. Dynamic deterrents like solar blinkers are inexpensive and initially effective but quickly lose impact due to habituation. Habitat and food management can divert wild boars but risk increasing populations if overused. Strict regulation is essential to avoid the “feeding paradox.”

#### 4.2.2. Control Strategies: Direct Effectiveness Accompanied by Complex Challenges

Control strategies directly manage wild boar populations but encounter practical and social challenges. In their native Eurasian range, where wild boars have co-evolved with human hunting, hunting remains the most immediate and effective method for population control. However, its effectiveness is constrained by hunter motivation, method efficiency, and regional coordination. Studies in Europe indicate that traditional, dispersed recreational hunting is insufficient to halt population growth [31]. Enhancing efficiency, such as by promoting coordinated driven hunts, and improving inter-regional cooperation are essential for better outcomes [34]. Meanwhile, non-lethal reproductive control methods (e.g., immunocontraception) offer theoretical potential but face significant economic and logistical challenges, remaining feasible only for small-scale, high-value experimental sites [31].

#### 4.2.3. Compensation Strategies: The Crucial Social Dimension

Compensation mechanisms are essential social safeguards that help maintain farmers’ tolerance. However, their success depends on transparent and equitable implementation. Bureaucratic delays and a lack of transparency often undermine straightforward financial payouts, potentially exacerbating social tensions [36]. Beyond direct financial redress, compensation can be innovatively linked to population control. For example, local communities can be empowered to hunt wild boars to offset crop losses with wild meat, or hunting licenses can be sold to external hunters. This approach aligns with the fundamental strategy of mitigating unavoidable damage through regulated hunting. Furthermore, participatory management approaches—such as multi-stakeholder collaborative decision-making facilitated by role-playing games—can significantly enhance the acceptance of mitigation measures by fostering dialog and consensus [39]. Nevertheless, these frameworks depend heavily on a supportive institutional environment and established trust [38].

In summary, mitigation strategies differ significantly in efficacy, cost, and sustainability. To facilitate an intuitive comparison, a systematic evaluation of these approaches based on the ECS framework are presented in Table 3 [26,30,31,36,38,39].

### 4.3. Multi-Strategy Synergy and Optimization Paths

Individual strategies have inherent limitations; therefore, a synergistic, multi-strategy approach is essential. Achieving this synergy requires precise spatiotemporal coordination. For example, combining electric fences for long-term protection with short-term acoustic and light deterrents during the crop maturation period effectively balances cost and efficacy [26]. Ecological phenomena, such as the “reserve effect,” can also be exploited to concentrate wild boar populations, facilitating more efficient control hunting [33]. Furthermore, intelligent monitoring networks enable a shift from passive response to active prevention by identifying conflict hotspots and tracking population dynamics [2,28].

Beyond technical measures, effective management requires integrating ecological controls with social governance. Crucially, optimization should follow two fundamental pathways based on land-use realities (1) where sufficient space can be reserved, providing large, undisturbed habitats for wild boars and restoring natural predators or implementing regulated hunting; (2) where space is inevitably shared, accepting some level of damage while offsetting losses through local hunting (compensating crop loss with wild meat) and selling hunting licenses to external hunters. Future efforts must tailor these strategy bundles to regional contexts (e.g., urban suburbs, agricultural zones, protected areas), enhance adaptive management through continuous monitoring, and integrate community capacity building to ensure sustainability.

## 5. Challenges and Prospects

### 5.1. Challenges

Although intelligent technologies such as infrared cameras and AI-based early warning systems are employed, their accuracy and coverage are limited by complex and variable ecosystems. For example, miniature biorecorders developed by Czech researchers can detect wild boar feeding behavior, but their accuracy declines sharply in dense vegetation or rugged terrain [1]. Additionally, these methods rely heavily on the manual equipment deployment, making large-scale, continuous monitoring costly and challenging. Advanced models, including LSTM time series algorithms, have been applied to predict wild boar invasion; however, their effectiveness in complex ecosystems requires be further validation and optimization [3]. Consequently, significant gaps persist in wild boar population monitoring techniques.

There is a lack of long-term assessment of wild boar control measures. Most studies focus on short-term effects, while long-term monitoring and evaluation remain scarce. For example, in Lower Saxony, Germany, electric fences effectively prevent wild boar invasions in the short term; however, their maintenance costs are high, and their protective efficiency declines over time [47]. Similarly, hunting strategies initially reduce populations, but their long-term ecological impacts and the risk of population rebound are insufficiently assessed [10]. Establishing a systematic, long-term evaluation framework is crucial for scientifically assessing control effectiveness and optimizing management strategies.

The existing literature exhibits bias that limits its generalizability. Most studies originate from Europe, North America, and East Asia, whereas conflict dynamics in Africa, South America, and Southeast Asia remain understudied. Publication bias favors positive outcomes, leading to an overestimation of strategy effectiveness, as few studies report null results or failures. Furthermore, the majority of research focuses on short-term outcomes (1–3 years), with long-term ecological and social impacts (≥10 years) rarely examined. Future research should include negative findings, extend monitoring durations, and broaden geographic coverage.

### 5.2. Policy Recommendations

**Figure 1:**
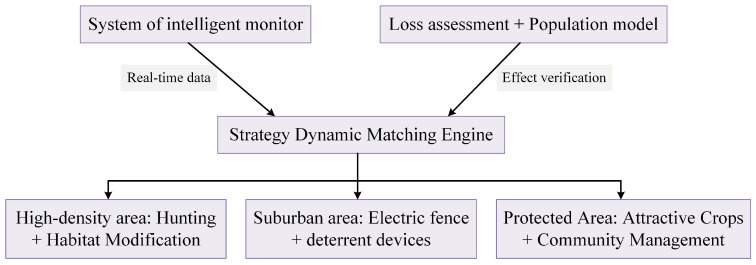
System of intelligent monitoring.

(1) **Multi-level management framework.** As shown in Figure 1, wild boar conflict management requires a multi-level management framework to adapt to different regional, ecological and socio-economic conditions, and a single policy is difficult to solve complex human–animal conflicts. Therefore, the “village-level monitoring county-level response” system in Zhejiang Province, China, and the game theory conflict-mitigation experiment in France can be used to construct a management framework at the national, provincial, municipal, and county levels [2,48]. The framework should clarify the responsibilities and authorities of all levels of government, enhance cross-departmental collaboration to ensure the effective implementation and supervision of policies, and encourage localities to formulate specific implementation plans based on actual conditions to form a governance system that is coordinated and efficient from top to bottom.
(2) **Innovate the compensation mechanism.** To alleviate the economic losses of farmers caused by wild boar conflicts, compensation mechanisms need to be innovated. Ecological insurance, as a means of risk diversification, could be considered for inclusion in the compensation system for wild boar damage. This includes providing wild boar damage insurance to farmers through government subsidies and the participation of insurance companies to reduce their economic burden. In addition, the carbon-trading linkage mechanism can also serve as one of the innovative compensation approaches. By quantifying the contribution of wild boar habitat protection to carbon sinks, we can convert ecological value into economic value and provide additional economic incentives for farmers [20]. The implementation of the innovative compensation mechanism requires the joint participation and coordination of the government, insurance companies and carbon-trading markets to form a diversified and sustainable compensation system.
(3) Context-specific strategy bundles. Instead of a one-size-fits-all approach, we recommend three bundles based on regional characteristics:
  ① High-density agricultural areas: Prioritize regulated hunting (with annual quotas and coordinated driven hunts) combined with electric fencing during crop maturation. Compensation funds should be linked to farmers’ adoption of preventive measures.
  ② Suburban interfaces: Deploy smart monitoring (AI cameras, vibration sensors) and physical barriers (e.g., solar-powered electric fences) together with public warning systems. Lethal control is often politically sensitive; focus on exclusion and aversive conditioning.
  ③ Protected areas: Maintain buffer zones of unpalatable crops, establish community co-management committees, and use ecological compensation to offset damage. In areas where predators are absent, regulated hunting or trapping can be allowed.

These bundles should be implemented iteratively, with monitoring data used to adjust measures annually.

### 5.3. Technological Innovation

(1) Intelligent early warning systems. Drones can quickly cover large areas, capture images of wild boar activities through high-definition cameras, and AI algorithms can analyze the image data in real time to identify wild boar populations, activity ranges, and invasion trends [11]. Therefore, an intelligent early warning system that combines drones with AI recognition is an important technical direction for future wild boar conflict prevention and control. The practical application of this system can not only improve monitoring efficiency but also provide a scientific basis for the formulation of prevention and control measures. For example, Seoul, South Korea, has attempted to use AI cameras and vibration sensors to monitor wild boar invasions in real time. In the future, drone technology can be further integrated to build a more complete intelligent early warning system to achieve real-time monitoring and early warning of wild boar activities [9].
(2) Gene regulation technology (e.g., gene drive) remains in early experimental stages and faces substantial technical, ethical, and regulatory hurdles. It is not currently a viable management tool for wild boar populations. Permitting processes have not begun in most countries. Future research may explore its potential under strict oversight, but practical application is unlikely in the near term.

## 6. Conclusions

This review synthesizes global evidence on mitigating human–wild boar conflicts. The main findings are threefold. First, no single strategy is universally effective; multi-strategy synergy (e.g., combining electric fencing with community-based hunting and compensation) consistently outperforms single interventions, showing nonlinear effectiveness gains. Second, the proposed ECS framework reveals that while preventive measures (e.g., electric fences) offer high technical efficacy, their economic feasibility and long-term sustainability are often low without institutional support. Third, critical knowledge gaps remain: most studies assess short-term outcomes only; long-term population and ecological impacts are rarely tracked; and monitoring technologies still lack accuracy and coverage in complex terrains. These gaps call for more rigorous, long-term evaluations and adaptive management frameworks.

The mitigation of conflicts between wild boars and humans requires moving beyond single-dimensional solutions. Sustainable mitigation demands integrating ecological monitoring, economic compensation, and social participation into an adaptive management framework. Practically, this means linking compensation funds to farmers’ active implementation of preventive measures, strengthening dynamic population monitoring to guide hunting quotas, and establishing participatory platforms where local communities co-design management rules.

From a parsimonious and practical perspective, two fundamental pathways exist for mitigating human–wild boar conflicts. First, where sufficient space can be reserved, wild boars should be provided with large, undisturbed habitats, and populations should be regulated by restoring natural predators or through regulated hunting. Second, where space is inevitably shared, societies must tolerate some level of damage while offsetting losses through practical mechanisms, such as local hunting (compensating crop loss with wild meat) and selling hunting licenses to external hunters. Our review demonstrates that successful cases (e.g., Zhejiang, France) combine elements of both pathways, whereas failures often result from neglecting the first pathway while inadequately implementing the second.

Future research must break down disciplinary barriers and promote the deep integration of ecology, sociology, and policy science. Resolving conflicts involving wild boars requires the collaborative efforts of multiple disciplines, including ecology, sociology, and policy science. Ecologists can provide technical support for population dynamics monitoring and ecological impact assessments, offering a scientific foundation for management decisions. Sociologists can analyze farmers’ behavior and social psychology to supply a social basis for policymaking and ensure the feasibility and effectiveness of the policy; Policy scientists are responsible for designing a scientific and rational policy framework, clarifying the responsibilities and authorities of governments at all levels, and strengthening cross-departmental collaboration and supervision. Through interdisciplinary collaboration, the scientific, systematic, and sustainable management of wild boar conflicts can be advanced, establishing a model characterized by government leadership, social participation, and technological support, thereby providing valuable references for global wildlife management.

## Figures and Tables

**Table 1:** Distribution characteristics of literature from 2000 to 2025.

Stages	Time Periods	Annual Average Literature Volume	Research Topic Focus
Slow Start period	2000–2010	≤4 articles	Population density monitoring, assessment of the effectiveness of basic protective measures (fencing)
Steady growth period	2011–2017	5–8 articles	Reproductive regulation, habitat management
Rapid outbreak period	2018–2023	Articles 9–14	Hunting management, gene regulation, and collaborative strategies
Adjust the pullback period	2024–2025	11–12 articles	Economic-ecological balance mechanisms and compensation system optimization

**Table 2:** Comparison matrix of case features.

Dimension	Features of Success Cases	Failure Case Defect
Governance subject	Multi-sectoral collaboration	Single-department dominance
Funding mechanism	Compound compensation (insurance + fund + livelihood)	Single compensation
Technology application	Localization adaptation (accuracy > 85%)	Standardized transplantation
Monitoring system	Smart networks	Manual statistics
Cultural fit	Cognitive intervention	Cultural exclusion

**Table 3:** Evaluation and comparison of major mitigation strategies.

Mitigation Strategy	Technical Efficacy	Economic Feasibility	Ecological Adaptability	Social Acceptability	Management Sustainability	Comprehensive Evaluation/Recommendations
**Electric fencing system**	High: Provides sustained protection during critical periods, with long-term cost-effectiveness superior to traditional fences.	Low: High initial investment and maintenance costs; challenges in complex terrain.	Moderate: May trigger behavioral adaptations (e.g., digging, shifting ranges).	Moderate: Varies; concerns over animal welfare and landscape esthetics in some contexts.	High: Durable infrastructure with stable, long-term protection.	**Recommended for long-term protection in high-value or high-risk areas, requiring sustained maintenance.**
**Dynamic deterrence devices**	Moderate (High initially, Low later): Significant short-term efficacy but rapid decay due to habituation.	High: Low initial cost, flexible deployment.	Low: Rapid habituation is the primary limitation; requires randomization of stimulus modes to delay habituation.	High: Low-tech, non-lethal, and easily accepted by the public.	Low: Short effective duration; high frequency of re-application or device rotation needed.	**Suitable for short-term, supplemental use or in areas with low damage intensity; must be combined with other measures.**
**Habitat and Food Management**	Moderate: Effectively diverts activity to buffer zones but risks of the “feeding paradox.”	High: Low cost, guided by ecological principles.	Variable (Low-Moderate): Excessive supplementary feeding may artificially increase population density; requires strict threshold management.	Moderate: May be controversial if perceived as “feeding pests”; requires clear communication.	Moderate: Sustainable if integrated into broader land-use plans; depends on ongoing monitoring and community cooperation.	**Applicable as a preventive measure in landscape planning; effectiveness hinges on precise implementation and monitoring to avoid counterproductive outcomes.**
**Hunting Management**	High: Most direct and effective population control lever.	Moderate: Variable; depends on hunter numbers, skill, and coordination; “reserve effect” offers strategic opportunities.	Moderate: Selective pressure can alter population structure (e.g., earlier reproduction); spatial distribution influenced by hunting pressure.	Low (High in hunter communities): Controversial; public attitudes vary widely between rural and urban groups.	Moderate: Relies on a shrinking demographic of hunters; institutional support and training are critical.	**Core tool for population control. Efficacy is maximized through coordinated, efficient hunting practices and integration with other strategies.**
**Reproductive Control**	Low (Theoretical): Potential for non-lethal, reversible control.	Extremely Low: High cost of capture and administration; infeasible for large-scale wild populations.	Low: Coverage challenges may render it ineffective; behavioral tolerance to disturbance complicates capture.	High: Non-lethal method with broad public acceptance in principle.	Low: High operational complexity and dependence on repeated interventions; not yet field-ready.	**Currently suitable only for small-scale, high-value experimental sites; not a viable mainstream alternative to hunting.**
**Ecological Compensation**	Low (as a deterrent): Compensates for economic losses but does not directly reduce damage.	Moderate: Funding costs can be substantial; depends on administrative efficiency.	Low: No direct impact on wildlife behavior or population dynamics.	Moderate (High among affected farmers): Essential for maintaining tolerance; however, bureaucratic processes can erode trust.	Low: Prone to inefficiency, fraud, and dependency if not paired with incentives for preventive action.	**An essential social safety net. Efficacy depends on transparent, timely, and scientifically designed implementation linked to proactive preventive measures.**
**Participatory Management**	Moderate (Indirect): Facilitates consensus and improves the design and execution of other strategies.	Moderate: Costs are related to organization and facilitation, not physical infrastructure.	High: Promotes solutions tailored to local social–ecological contexts.	High: Fosters trust and ownership among stakeholders; mediates deep-seated conflicts.	High: Builds local capacity for ongoing, adaptive management.	**A crucial foundational approach. Enhances the efficacy and sustainability of all other strategies by ensuring they are socially acceptable, context-specific, and collaboratively implemented.**

## Data Availability

No new data were created or analyzed in this study.
